# OWLing Clinical Data Repositories With the Ontology Web Language

**DOI:** 10.2196/medinform.3023

**Published:** 2014-08-01

**Authors:** Raimundo Lozano-Rubí, Xavier Pastor, Esther Lozano

**Affiliations:** ^1^Hospital ClínicUnit of Medical InformaticsUniversity of BarcelonaBarcelonaSpain; ^2^Autonomous University of BarcelonaDepartment of Computer ScienceBarcelonaSpain; ^3^Universidad Politécnica de MadridOntology Engineering GroupMadridSpain

**Keywords:** biomedical ontologies, data storage and retrieval, knowledge management, data sharing, electronic health records

## Abstract

**Background:**

The health sciences are based upon information. Clinical information is usually stored and managed by physicians with precarious tools, such as spreadsheets. The biomedical domain is more complex than other domains that have adopted information and communication technologies as pervasive business tools. Moreover, medicine continuously changes its corpus of knowledge because of new discoveries and the rearrangements in the relationships among concepts. This scenario makes it especially difficult to offer good tools to answer the professional needs of researchers and constitutes a barrier that needs innovation to discover useful solutions.

**Objective:**

The objective was to design and implement a framework for the development of clinical data repositories, capable of facing the continuous change in the biomedicine domain and minimizing the technical knowledge required from final users.

**Methods:**

We combined knowledge management tools and methodologies with relational technology. We present an ontology-based approach that is flexible and efficient for dealing with complexity and change, integrated with a solid relational storage and a Web graphical user interface.

**Results:**

Onto Clinical Research Forms (OntoCRF) is a framework for the definition, modeling, and instantiation of data repositories. It does not need any database design or programming. All required information to define a new project is explicitly stated in ontologies. Moreover, the user interface is built automatically on the fly as Web pages, whereas data are stored in a generic repository. This allows for immediate deployment and population of the database as well as instant online availability of any modification.

**Conclusions:**

OntoCRF is a complete framework to build data repositories with a solid relational storage. Driven by ontologies, OntoCRF is more flexible and efficient to deal with complexity and change than traditional systems and does not require very skilled technical people facilitating the engineering of clinical software systems.

## Introduction

The health sciences, particularly medicine, are based upon information and communication. Clinical practice and research processes consist mostly of collecting data, summarizing this data, and using information derived from the data. This information, properly integrated with clinical knowledge, constitutes the base for decision support and generation of new knowledge. Nevertheless, in spite of great advances in the information and communication technologies (ICT) domain during past years, the progress in medical informatics is slower than predicted. Clinical information systems are failing to provide true support for clinicians’ needs [1,2]. Although there is a broad commercial offer of clinical information systems to support patient management and the electronic patient record (EPR), they are focused primarily on the economic and administrative processes, and lack the needed functionality to manage clinical data. Existing central data warehouses usually fail to support the creation of structured variables for research use [3], so it is necessary to build dedicated systems [4]. As a result, there is little institutional support within health organizations for the collection of clinical data, especially for research.

The implementation of research data repositories has been reported to increase the capacity of a research team [3]. Some surveys show that individual organizations are progressing to the development, management, and use of clinical repositories as a means to support a broad array of research [5]. Although most researchers already use some software system to manage their data, there continues to be widespread use of basic and general-purpose applications, such as spreadsheets, and additional support has become necessary for managing datasets. Interestingly, the barriers to acquiring currently available tools are most commonly related to financial burdens [6].

This is the situation in the Hospital Clinic of Barcelona, which has a long tradition in biomedical research and stands as a benchmark institution both nationally and internationally [7,8]. A research project cannot be understood now without ICT support to some extent. Nevertheless, the spreadsheet remains the key tool for research data management because financial limitations restrict the acquisition of more complex tools. Continuous change is a characteristic of the biomedical domain, and building applications that can handle it is very expensive.

We have developed Onto Clinical Research Forms (OntoCRF), a framework for the definition, modeling, and implementation of data repositories. Most importantly, OntoCRF is capable of meeting change at a minimal cost because the implementation of a new repository in OntoCRF does not need additional database design or programming. All information required to define a new project is explicitly declared in ontologies, reducing the time and cost of development compared to traditional solutions. The repositories implemented with OntoCRF are accessible via a website for data entry, thus facilitating the collection of distributed data.

## Methods

### Background

The Hospital Clinic of Barcelona has a growing need for systems for the collection of clinical data. The Medical Informatics unit at Hospital Clinic of Barcelona has experience designing and implementing databases for research [9-11]. Some general requirements for data management reported in the literature [3,5,12] are as follows:

1. The ability to efficiently acquire, store, and manage large volumes of structured data, preferably in a centralized repository.
2. To provide a Web interface for researchers to allow them to have a distributed access to the data in order to introduce new data or to retrieve existing data. Data are usually gathered by various researchers, often in different locations.
3. Data security, including access control, to assure the persistence of the data.
4. To facilitate the access to the data, including researcher “self-serve” access.
5. To be able to easily accommodate changes in the structure of the data, minimizing service disruption when such a model change occurs.

The Hospital Clinic of Barcelona has used an EPR system since 1995. Three different commercial systems have been used during this time, the last one including a data warehouse, but they were primarily focused on economic and administrative processes. Although these systems allowed gathering of some limited clinical data, none of them were intended to register additional data.

Because of financial limitations, there has been widespread use by researchers of basic and general-purpose application software, such as spreadsheets. The same situation is reported by other authors [3,6]. The use of general-purpose application software has serious drawbacks: an unfriendly user interface, few guarantees for maintaining the consistency of data, difficulties in sharing and consolidation of data, and limited ability to exploit data. Desktop application software programs are definitively not designed to meet the above mentioned criteria.

When there is an adequate budget available, it is possible to build a more sophisticated system. Usually, these systems are built using a multitier architecture composed of a centralized database, an application server, and a Web server providing the user interface. However, this architecture presents some disadvantages. First of all, the development of such applications is a laborious task, as is their extension to accommodate changes. Consequently, this approach is not suitable for domains where data and model evolution is the norm [12]. Secondly, this classical approach requires a very specialized panel of computer technicians and this often leads to communication problems between the biomedical researchers and the development team. Thirdly, the development cost and the cost of information technology (IT) personnel require a high investment [6] sometimes for a short project time (research projects typically last 2-3 years). Finally, using this kind of approach within a large organization produces applications very different among them, and the distribution of data across multiple sources, which complicates the ability of researchers to use the data for answering their research questions [4].

These considerations—the lack of available tools in our organization and the disadvantages of traditional database systems—prompted us to seek an alternative and to build a platform to deploy research projects and clinical registries.

The advances in knowledge management tools and methodologies in previous years provided the opportunity for a new approach. Ontologies as explicit conceptualizations of a domain [13] seem well adapted to the task of representing medical data. Ontologies resemble databases from an operational perspective because they can be populated with instance data and deployed as parts of information systems for answering queries [14]. Languages to represent ontologies, such as Ontology Web Language (OWL), are designed to be extensible and able to accommodate model changes. The flexibility of ontologies is a major advantage of the technology [14]. These characteristics make ontologies suitable to build a conceptual platform on which specific applications can be deployed [12].

In addition, the use of ontologies is more and more common in the health care field [15-21], which provides an environment to seamlessly integrate the new information models with existing ontologies.

### Use Case Presentation

In the following, we will use examples from current projects to illustrate how the system works. One registry is the European Forum on Antiphospholipid Antibodies, a registry of patients with catastrophic antiphospholipid syndrome (CAPS). This project aims to establish an international dataset of all diagnosed patients with CAPS. For each clinical case, the following data are registered: demographic data, previous clinical manifestations, precipitating factors, clinical findings organized by organs, laboratory results, and treatment followed.

### Outcome

**Figure 1:**
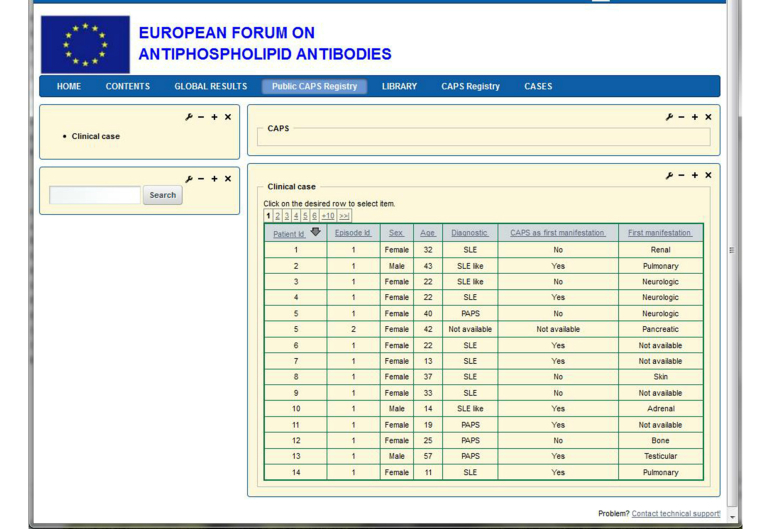
List of clinical cases within CAPS registry.

**Figure 2:**
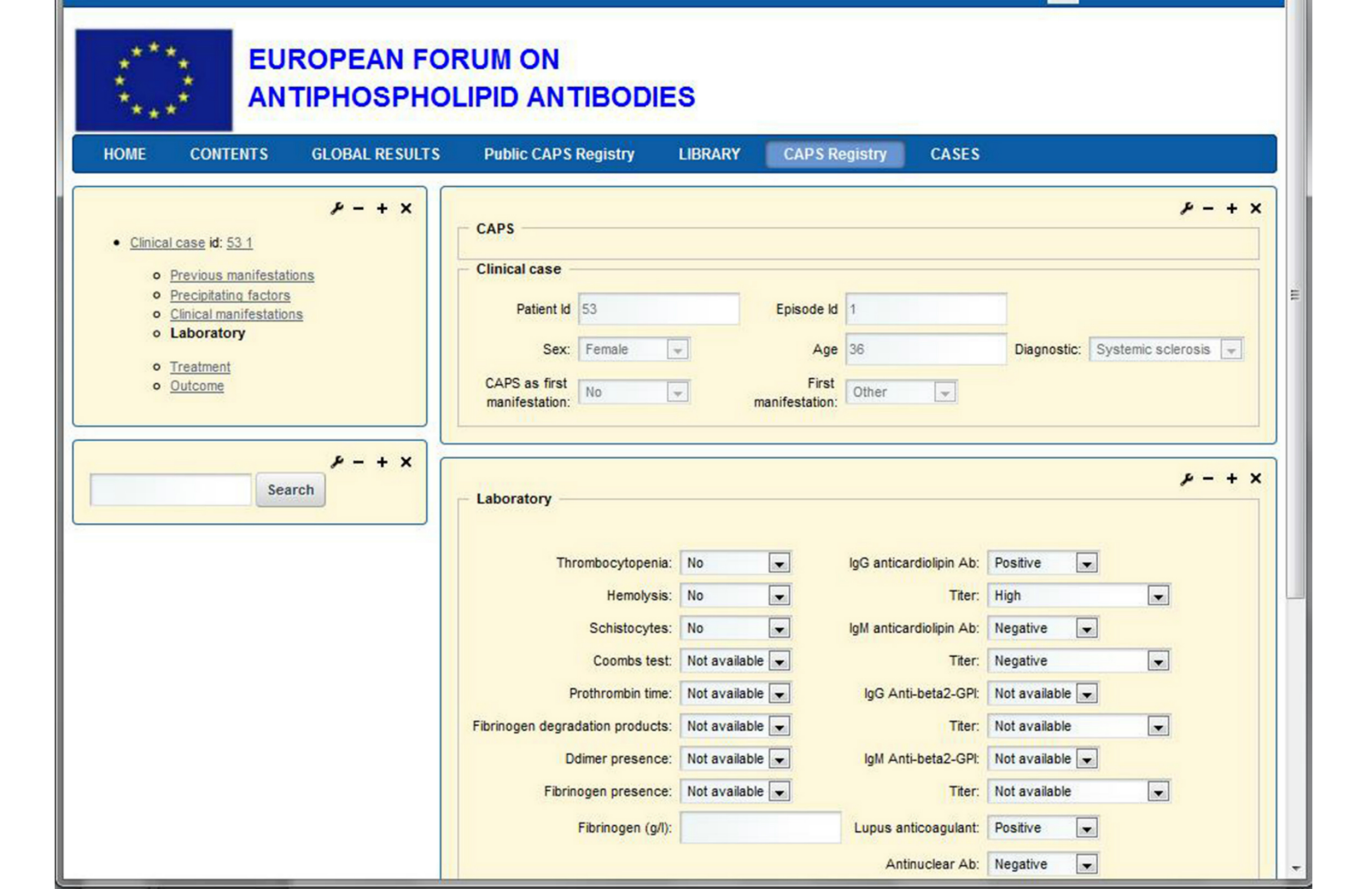
A CAPS registry clinical case with laboratory results.

The data have to be stored in a centralized database to allow periodic statistical analyses on them. In order to allow a decentralized introduction of data, a Web-based application program is needed. Screenshots of the data entry screen are shown in Figures 1 and 2. Figure 1 shows the list of clinical cases from the CAPS registry and Figure 2 shows a concrete case with some laboratory results.

The panel on the top left allows for navigation through the different parts of the registry. The windows on the right, which constitute the formularies to fill in, are composed of single cells, combo boxes, check boxes, radio buttons, etc, to introduce and visualize the data.

### Proposed Solution

OntoCRF is a framework to build clinical data repositories initially designed for research. The general idea of OntoCRF is to combine the best of two technologies: the expressivity and flexibility of ontologies with the proven robustness and efficiency of relational databases. Previous work by our team has already demonstrated the feasibility of using a relational persistence layer to store ontologies [22,23].

As a general requirement, all information needed for the system to work should be modeled in ontologies. Furthermore, no additional programming should be necessary to implement a new project. By doing so, each different project has a different ontology that models both the data and the user interface. The ontology indicates which data are needed (eg, age, sex) and how to represent them on the screen (ie, a single cell in the first row, a radio button in the second row). The program code should be the same for different projects, but is capable of “interpreting” the corresponding ontology to implement different projects.

Although prior work was done with Resource Description Framework (RDF), we choose OWL [24] as the modeling language. The justification of using OWL is twofold:

1. Able to reuse existing ontologies. For example, the ontologies stored in BioPortal [25], many in OWL format, are accessible from Protégé.
2. Able to make automatic reasoning in the future. Although not explored yet, we have plans to use reasoners such as Pellet [26] for consistency checking, automatic classification, etc.

OWL is a standard with wide support in the Semantic Web community. Thus, tools developed by the Semantic Web community can be directly applied to the data, such as Protégé [27], as an ontology-editing tool. The election of Protégé is motivated by our previous work on relational support for ontologies [22]. The persistence layer for both models and instantiated data are provided by a relational database.

**Figure 3:**
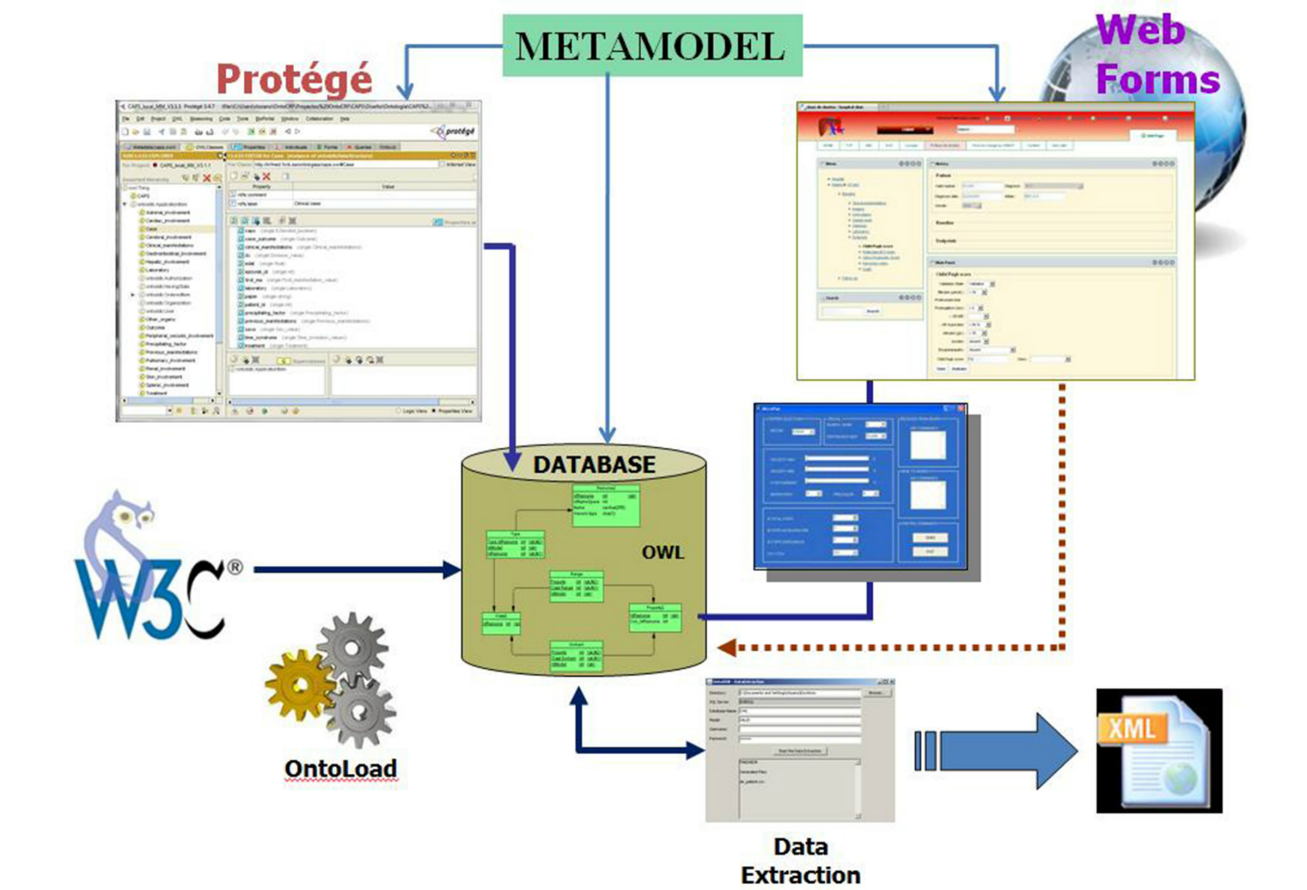
General architecture of OntoCRF.

OntoCRF is composed of the following modules: (1) a relational database for storing the ontologies and instantiated data, (2) an ontology editor based on Protégé, (3) a graphical user interface (GUI) based on Liferay [28], (4) a metamodel describing the primitives of the system, (5) an application for data extraction in the back end, and (6) an application for ontology upload in the back end. The general architecture is shown in Figure 3.

### Storage of Ontologies and Instantiated Data

OWL database (OWL-DB) is a relational database used for storing ontologies and instantiated data, following an approach similar to the Entity-Attribute-Value (EAV) schema. EAV schemas allow for changing the data structure and have proven their utility for clinical applications [29,30] The database was designed according to the OWL specification [24]. Based on Theoharis [31], storage schemes can be classified as schema-oblivious (1 table is used for storing the statements), schema-aware (1 table per class or property is used), and hybrid (1 table per metaclass and property instances with different range values is used).

In OntoCRF, the chosen storage architecture is basically a hybrid model, which is the model that achieves the best performance according to Theoharis [31]. In OWL-DB there is a table for each OWL metaclass, such as resource, class, property, domain, and range. The values of property instances are stored in a table according to its range (eg, resource, string, integer). An identity-based approach is used to identify resources because the use of shorter identifiers versus long internationalized resource identifiers (IRIs) results in space and performance benefits [32].

An additional single table is used to store all triples defining the ontology. Adding or deleting statements in this table causes triggers to fire and thus update the rest of the tables. The statements table serves as interface with other applications. Any application able to manage OWL statements (eg, ontology edition tools) can be potentially connected with OWL-DB.

Furthermore, this approach has all the advantages of EAV schemes. Instead of specific tables for storing patient data, laboratory data, etc, there are tables representing the elements of OWL specification. Therefore, schema evolution can be easily supported. Whereas the addition/deletion of a new property requires the addition/deletion of a table in schema-aware approaches [31], it only requires the addition/deletion of rows in the hybrid model. As a result, neither the design nor the structure of the database needs to be changed for different applications.

**Figure 4:**
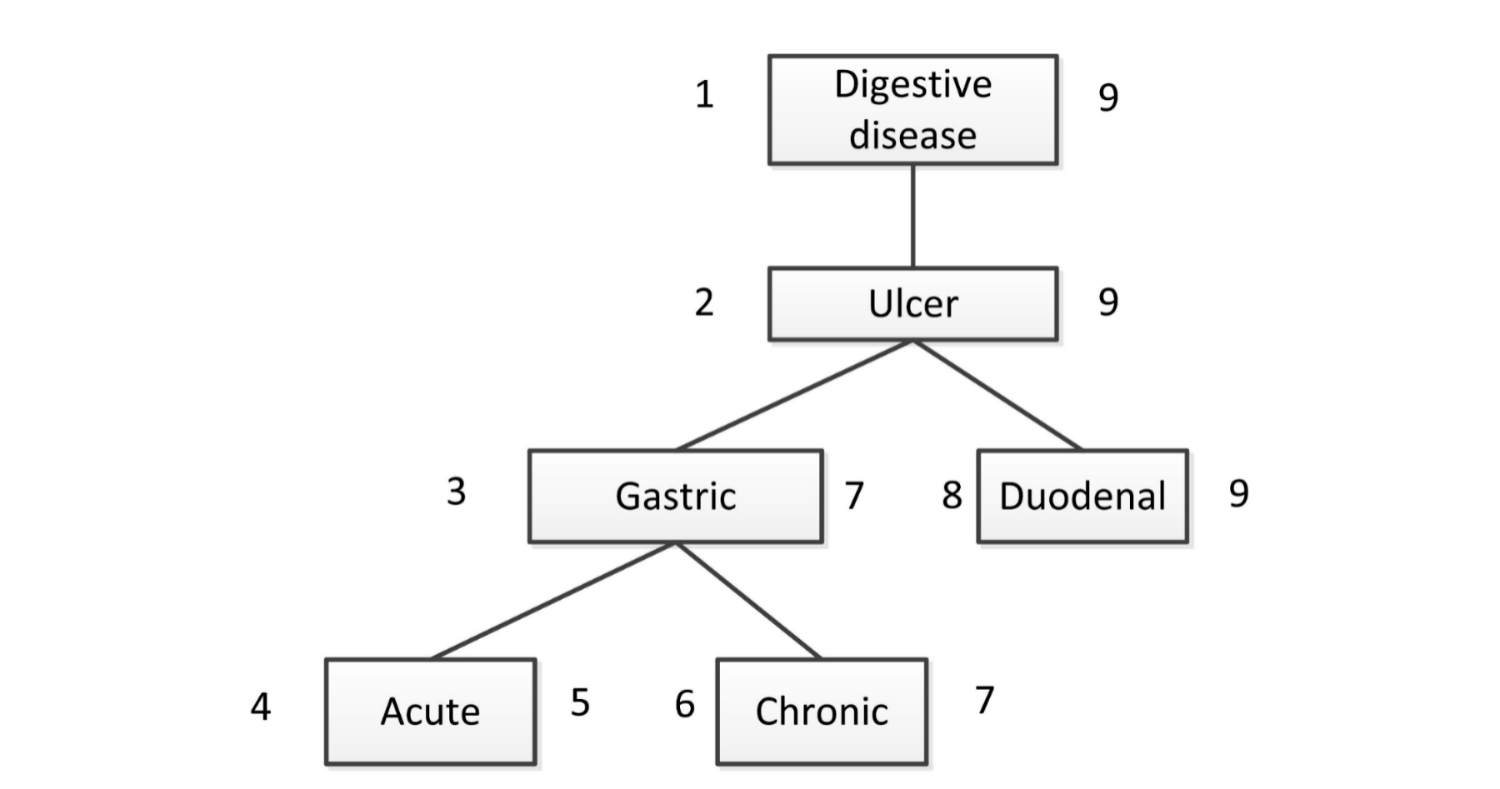
Example of a nested-set model of trees.

The design of the database is intended for a quick recovery of concepts through hierarchies of classes and subclasses. When only using a statements table, finding the subclasses of a class (through a variable number of levels) is a recursive problem, difficult to solve in the relational environment. To avoid this limitation, subsumption relationships between classes and properties are stored in specific tables, following a nested-set model of trees [33]. In this model, each node of the tree is labeled with two numbers (left and right), as shown in Figure 4.

Finding all subclasses of a given class (eg, digestive disease) becomes a very fast process: they are all classes with the right index (or left) comprised between the values ​​of the indexes of the class. Thus, all concepts defined as subclasses of it, such as acute gastric ulcer in the example, will be recovered in a very efficient manner, regardless of what level of depth in the hierarchy they are defined. Nevertheless, this design makes the management of multiple inheritance difficult. Currently, we duplicate the node with multiple inheritances in the class hierarchy, which represents only a small cost in storage space.

Other applications can interact with OWL-DB using an application programming interface (API) built with stored procedures. A set of functions retrieves the subclasses, properties, and instances of a named class, domain and range of properties, values of instance properties, etc, to extract information from the database.

The system can store all imported ontologies in the same database, maintaining the import relations between different ontologies.

### Ontology Authoring

The edition of the ontology is based on Protégé [27]. Protégé is a recognized standard for ontology edition, with more than 200,000 registered users around the world, and able to edit OWL ontologies. An interesting characteristic of Protégé is its extensibility capability. It is possible to include new functionalities to the tool by adding new plug-ins.

**Figure 5:**
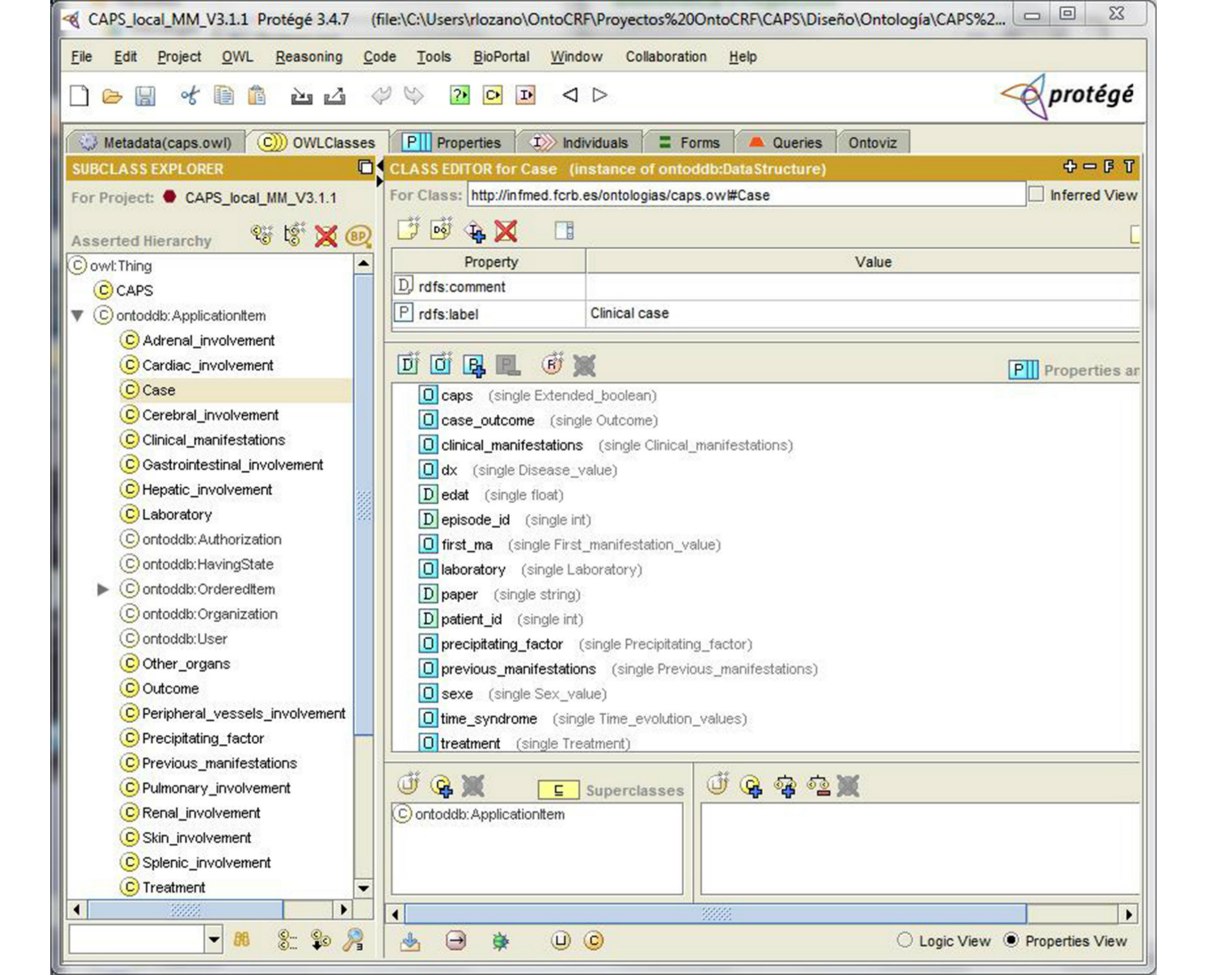
Ontology edition with Protégé.

With OntoCRF, the data to be registered are modeled in an ontology. To simplify and parallel relational databases, tables become classes and columns become properties. Figure 5 shows a snapshot of the CAPS ontology. Some classes representing the main groups of data to be registered (eg, case, Precipitating_Factors, Previous_Manifestations, Adrenal_Involvement, Cardiac_Involvement, laboratory, treatment) can be identified.

OWL and Protégé support additional functionality because the subclasses, metaclasses, etc, together with the metamodel allow Protégé to be used as a twofold design tool: (1) a kind of database design tool to define the data, its structure, and properties and (2) a graphic interface design tool to define how the data will be presented to the user.

**Figure 6:**
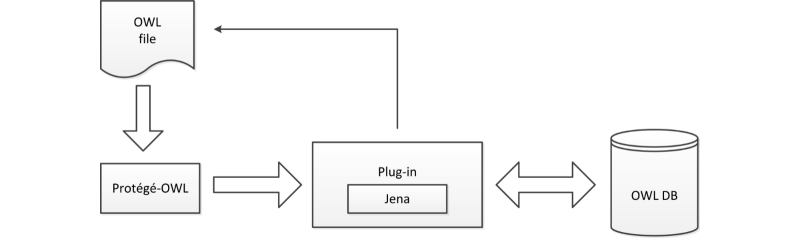
OWL-DB plug-in.

A plug-in developed by us, OWL-DB plug-in (Figure 6), connects Protégé with the OWL-DB module at the storage level. The OWL-DB plug-in uses Jena [34] to manage OWL statements and to communicate with OWL-DB.

The plug-in is a backend plug-in. This plug-in consists of a single class, which is subclass of the KnowledgeBaseFactory class provided by Protégé. It communicates by updating the statements table, which triggers the update of the rest of the tables in the database.

By using the OWL-DB plug-in, it is possible to load an ontology that was previously stored in the database to be edited in Protégé. The connection parameters provided are database management system (DBMS), server Internet Protocol (IP) address, database name, username, and ontology namespace. After changes are made in the ontology with Protégé, the user can choose either saving in the database only the last changes made or replacing the ontology entirely. If the ontology is importing other ontologies, an option is available to save all imported ontologies in the database at the same time.

By using the OWL-DB plug-in, an already existing OWL file in Extensible Markup Language (XML) format can be uploaded to the database. This is done using the Protégé menu option “Convert Project to Format...” where an option is available to choose the OWL-DB format. When storing ontologies in OWL-DB from Protégé, a local copy in an OWL file in XML format is automatically generated.

### The Metamodel

**Figure 7:**
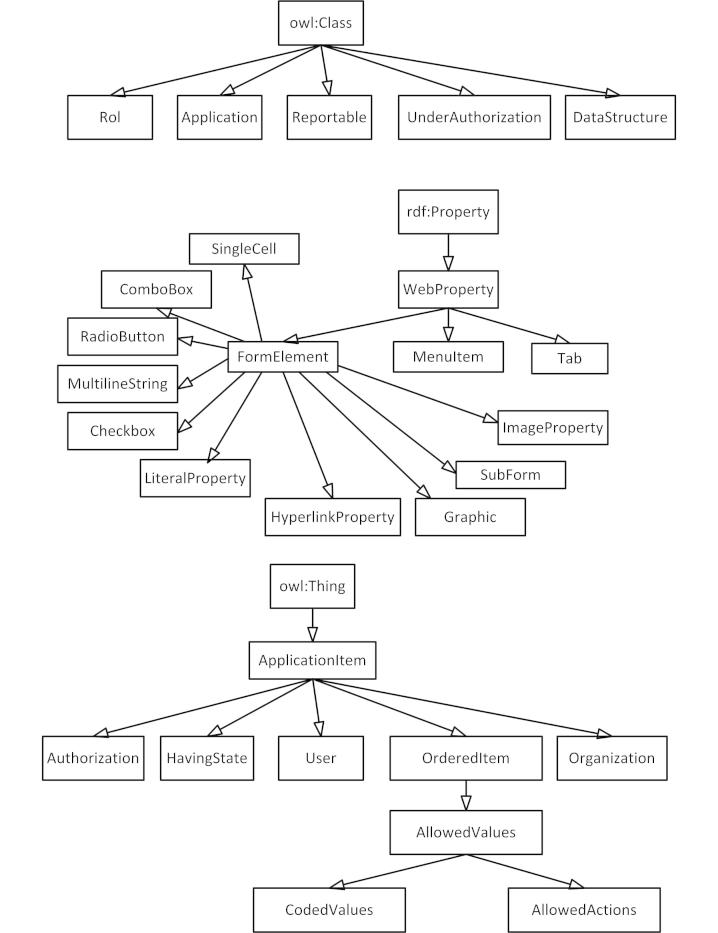
The ontology-driven database metamodel (OntoDDB-MM).

Ontology-driven database metamodel (OntoDDB-MM), the OntoCRF metamodel, is an ontology composed of a set of metaclasses, classes, and properties that define the available elements that can be used to build an application. These elements are recognized and used by the portlets to create the GUI. Figure 7 shows the main hierarchies.

In this metamodel, an application is represented by an instance of the metaclass application. In the CAPS registry example, the application is represented by the class “CAPS.” The different forms are represented by instances of metaclass DataStructure (eg, case, Previous_Manifestations, Clinical_Manifestations). At the same time, these classes are subclasses of the ApplicationItem class.

**Figure 8:**
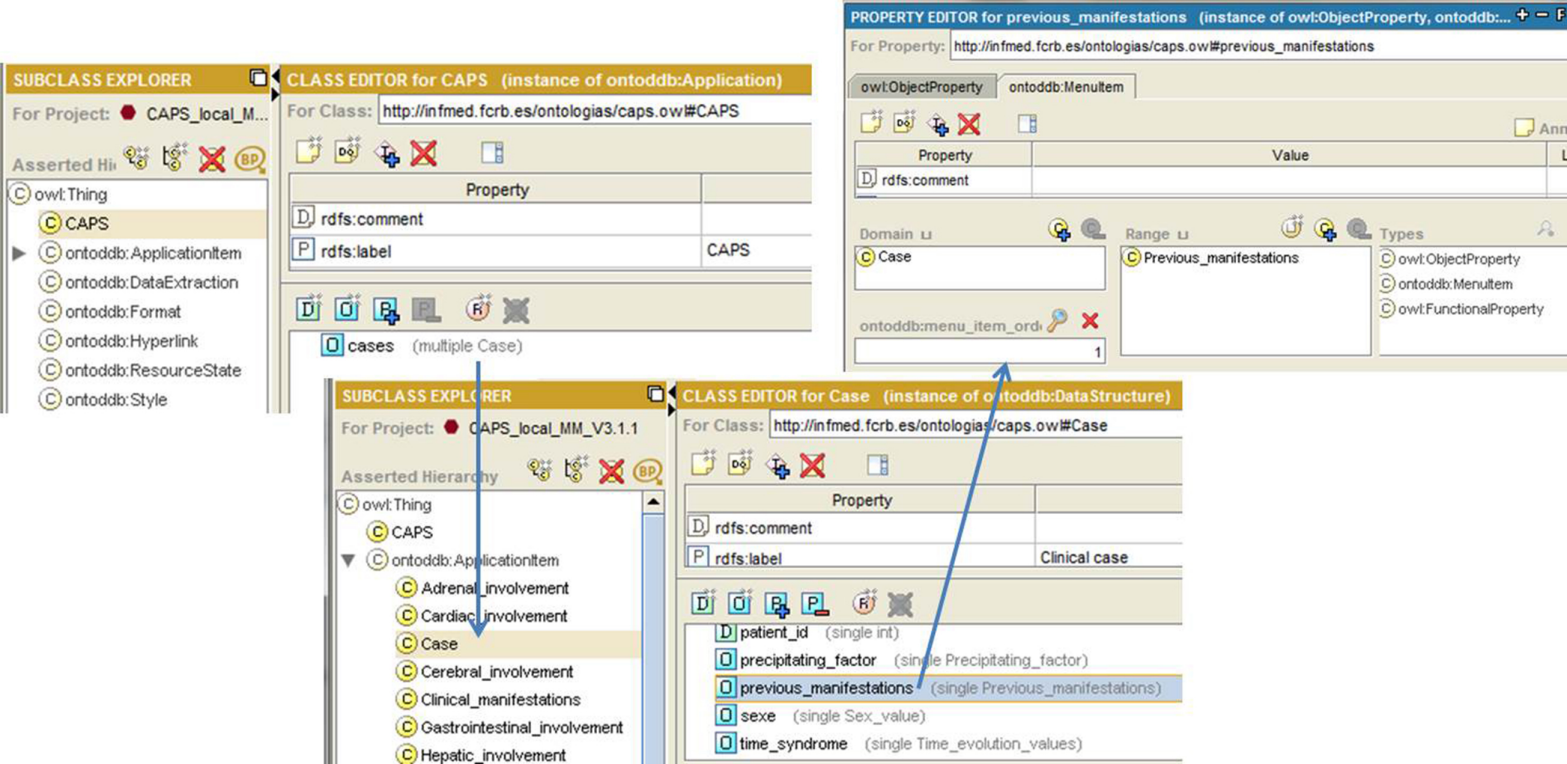
Example of menu elements.

A DataStructure can have several properties; some are DatatypeProperties and others are ObjectProperties. In our example, the properties Previous_Manifestations, Precipitating_Factors, Clinical_Manifestations, etc, are instances of both ObjectProperties and MenuItem. On the one hand, they are properties linking Case with other data structures and, on the other hand, they are menu elements. Figure 8 shows an example.

Each form field is an instance of one of the subclasses of FormElement, which determines its behavior: Checkbox, Combobox, Graphic, HyperlinkProperty, ImageProperty, LiteralProperty (to represent literals, do not expect a value), MultilineStringProperty, RadioButton, SingleCell, Password, and SubForm (not implemented yet).

To manage the form fields, the FormElement metaproperty introduces the following facets: webColumn (the relative column in the form where the field will be shown), webRow (the relative row in the form where the field will be shown), webDescriptionProperty (a flag to mark fields that are part of the description of the corresponding object and are shown in the headers, list, etc), webMandatoryProperty (a flag for fields do not allowed to have a null value), webIdProperty (a flag to mark fields that constitutes the Id of the corresponding data structure meaning that is mandatory to fill in the field and that the value must be unique), webEditionDisabled (a flag to avoid a field be edited), and webDirectlyDependent (a flag to identify depending objects). The objects that are values of webDirectlyDependent properties cannot exist without the object that has this property.

The metamodel indicates that there are constraints on the values to be used within each field. This is done by creating a subclass of the class AllowedValues for each field to be constrained. This class is a subclass of OrderedItem and the instances can have a relative order between them. If the subclass CodedValues is used instead of the class AllowedValues, each of the different options can have an attached code. This mechanism is similar to the method used by Rector et al [35] to constrain the codes to placeholders.

As an additional feature, the system can notify to specific users via email about the creation of new instances. This is useful to notify about adverse events, for example. To do that, the class whose instances have to be notified has to be a subclass of the Reportable metaclass.

Other classes, such as Role, UnderAuthorization, Organization, and Authorization, manage access permissions to the different resources, but they are only partially implemented at the present moment.

### The Graphical User Interface

Using Protégé and OWL-DB is enough to instantiate the ontology in a centralized repository. However, this would not be a suitable interface to an end user.

The user interface is built with portlets based on Spring model-view-controller (MVC) and deployed in Liferay. The business and controller levels are supported by Spring and the view level by JavaServer Pages (JSP) with JSP Standard Tag Library (JSTL). The screen presentation and direct interaction is made with HTML, Javascript, and JQuery. With this approach, the end user only needs a Web browser to interact with the system.

**Figure 9:**
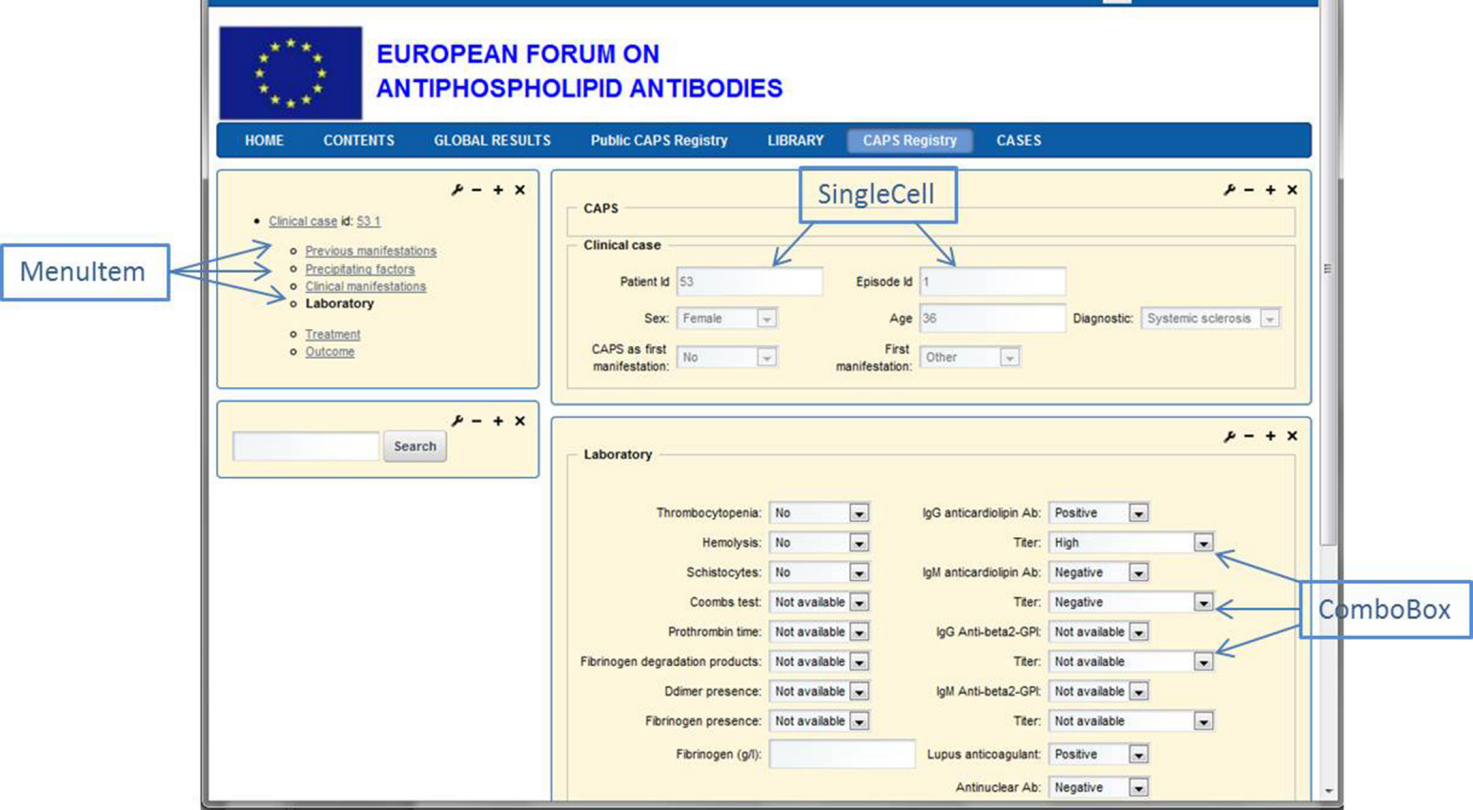
Example of form elements.

The GUI is created dynamically. The navigation menu, components generation, and all objects in general are created dynamically following the specification of the ontology. The portlets access directly to the OWL-DB stored procedures. Then the information about the application, expressed in the ontology, is used to build the Web pages on the fly, as shown in Figure 9.

### Data Extraction

The data extraction module allows periodic extractions of stored data for analyzation. This is done by invoking a Java application that will ask the user to provide the connection parameters. The output of this application is a set of XML files containing the data. These files can be imported to a conventional relational database or a statistical package to be analyzed. The data to be extracted is defined in the ontology as instances of the class DataExtraction. This class allows the user to specify which class of the application should be extracted and whether the value of their object properties must be traversed recursively or not.

Another available functionality can transform the entire ontology in a relational database. In this case, the output is a SQL script. This functionality can be used on a daily basis to maintain a relational version of the data.

### OWL-DB OntoLoad

OWL-DB OntoLoad is an application to directly upload an OWL ontology in XML format to the server by feeding the statements table directly instead of uploading it through the editor tool.

### Evaluation

To evaluate the usability of OntoCRF, the System Usability Scale (SUS) score was selected [36]. Developed in 1986 by Digital Equipment Corporation, it is a simple method to gauge first impressions of the appropriateness of software developments of end users. It consists of a questionnaire with 10 items:

1. I think that I would like to use this system frequently.
2. I found the system unnecessarily complex.
3. I thought the system was easy to use.
4. I think that I would need the support of a technical person to be able to use this system.
5. I found the various functions in this system were well integrated.
6. I thought there was too much inconsistency in this system.
7. I would imagine that most people would learn to use this system very quickly.
8. I found the system very cumbersome to use.
9. I felt very confident using the system.
10. I needed to learn a lot of things before I could get going with this system.

The answer to each item is a value from 1 and 5 (1=strongly disagree; 5=strongly agree). The results are computed following an algorithm that gives a unique result (SUS score) from 0 to 100.

The data from the questionnaires were entered into a database and analyzed using SPSS 21 statistical package (IBM Corp, Armonk, NY, USA).

## Results

OntoCRF has been used in more than 10 different projects. In general, these projects fall into one of the following categories:

1. Research projects with limited duration: a set of data, previously agreed, is collected and analyzed at the end of the project.
2. Clinical registries without a predetermined end date to modify the data collected during the project.
3. Implementation of clinical questionnaires.
4. Nonclinical applications.

The number of cases by project varies between a few hundred to 2000, with approximately 60 to 600 variables per case.

Table 1 shows a summary of the characteristics of the main projects running currently. The upload and download was made between Protégé 3.5 and OWL-DB, and refers to the entire ontology. To measure upload and download times without being influenced by traffic on the Internet, a local server was used with the following characteristics: SUSE Linux Enterprise Server 11 (x86_64) operating system, GNU/Linux 2.6.32.43-0.4-default x86_64 1 x Intel Xeon CPU E5-4640 0 @ 2.40GHz CPU, and 4Gb RAM.

**Table 1:** Summary of characteristics of the projects implemented.

Project	Number of statements	Disc space (Mb)	Number of classes	Number of properties	Number of instances	Upload time (sec)	Download time (sec)
1	102,371	48	147	365	26,414	65	12
2	103,652	72	91	288	8794	58	24
3	191,487	90	81	171	26,408	112	24
4	200,509	98	15,982	90	15,595	311	80
5	264,636	126	258	632	32,317	200	27
6	131,926	74	145	623	7553	75	17
7	79,350	47	125	535	5378	44	9

In all projects, OntoCRF has been able to meet their specific requirements and to cope with the requirements of modifications during the lifecycle of the projects. The modular architecture of the metamodel has proven its feasibility to accommodate new extensions of the system. Also, the separation of data layer and presentation layer allows the progressive addition of new functionalities as needed.

The flexibility provided by the system facilitates to provide prototypes from the initial moment, which is a very valuable resource for developers to work close to the physicians. From the beginning of the project, key users have material to work with, and it is possible to make online modifications and check results immediately.

**Figure 10:**
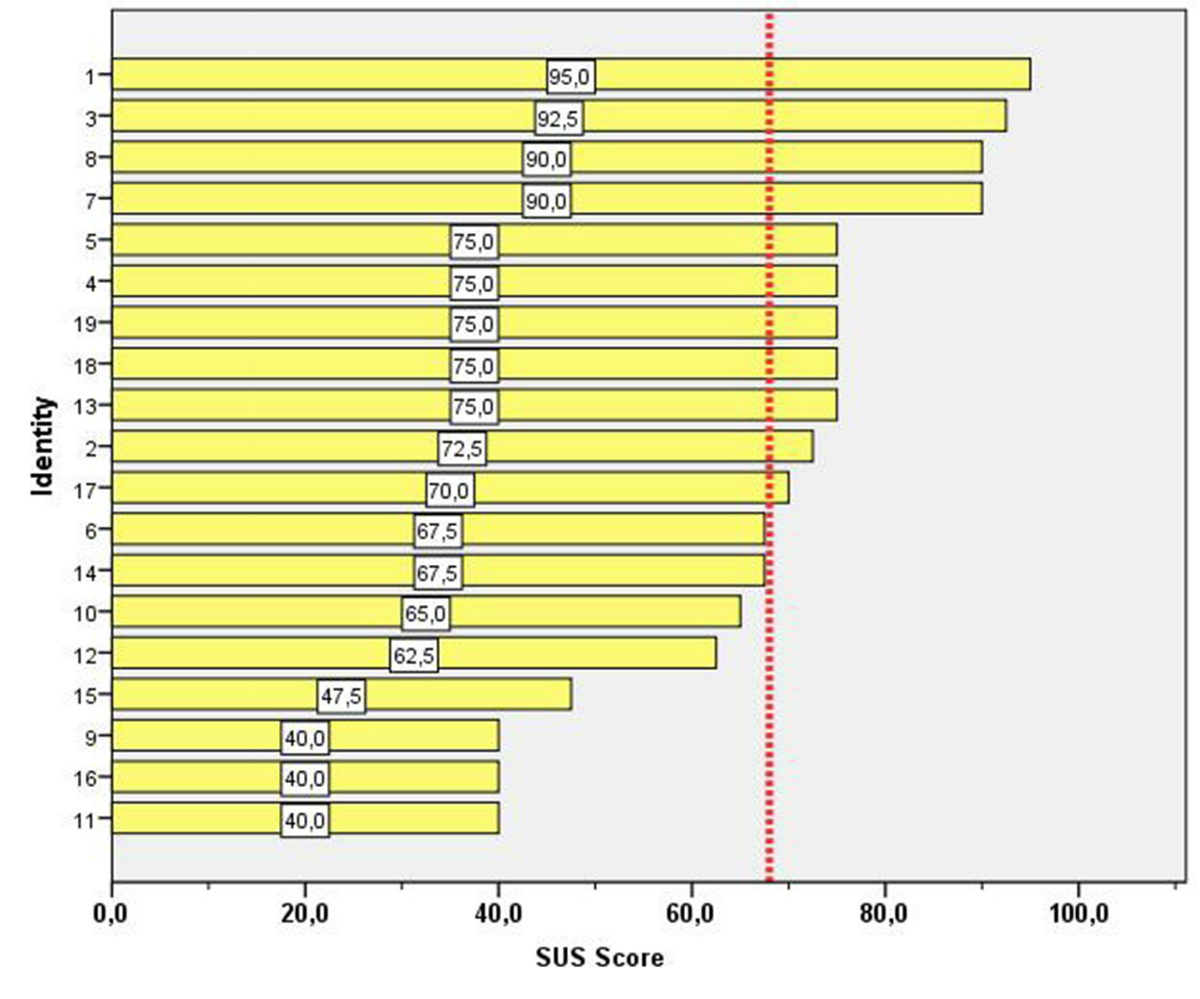
Results of the computed SUS score by respondent. Results are displayed in ascending order. The dotted line marks a score of 68 (above average).

A survey was distributed to a sample of 35 OntoCRF active users who used the system on a daily basis. Of these 35, 19 (54%) answered the questionnaire. Data were introduced into a database and the SUS score was computed. The results are displayed in Figure 10.

Of the 19 respondents, 11 (58%) computed a global SUS score greater than 68 which is recognized as “above average” [37]. According to Bangor et al [38], it is possible to grade over a curve based on the distribution of all scores in relationship with their quartile position. In all, 4 users (21%) gave the solution an A grade (excellent), 5 (26%) gave a C grade (good), 6 (32%) gave a D grade (pass), and 4 (21%) rated the solution with an F grade (fail) [38]

Because of the success achieved with OntoCRF in the first projects, which were primarily research projects, OntoCRF is currently being marketed and used in new types of projects.

## Discussion

### Overview

The focus of OntoCRF is to assist with data collection during research studies, automating the process as much as possible and minimizing the technical knowledge required from the final users for the creation and management of new studies. In particular, we provide an automatic system for dynamic creation of Webs driven by ontologies and with additional tools for the extraction and analysis of the data.

Our system, unlike other solutions, does not work with triples or RDF graphs; it works with ontologies, particularly those represented in OWL. Ontologies are stored in a relational database directly in OWL, following a hybrid approach. This eases the querying process because there is no OWL-SQL mapping needed. For instance, to retrieve the classes, the system just accesses the “class” table. Further logic is not necessary and it can all be done through simple SQL queries. Because we have to deal with very large ontologies, performance was a critical feature from the beginning; this OWL-driven approach achieved our efficiency requirements, whereas other systems failed.

Regarding the performance of the system, its behavior is quite linear. As Table 1 shows, any of the variables considered has a preponderant influence. In general, the upload and download times are proportional to the number of statements. The greater complexity of some ontologies, expressed by a higher proportion of classes and properties in relation to the number of instances, involves a slight penalty. Project 4 (with 2 orders of magnitude more in number of classes) showed a worse performance, but less than 4 times worse than other projects with a similar number of statements. This is due to the cost of maintaining the class hierarchy tree in the database, primarily when uploading the ontology. In previous versions of the system, each time a class was inserted in the database, all the indexes of the class hierarchy were recalculated. Project 4 showed the lack of scalability and efficiency of this approach; the system was not able to recalculate the indexes and remained working without end. In the current version, the entire class hierarchy is calculated only once after all classes have been inserted into the appropriate table. This approach represents only a gain of 2-3 seconds for the rest of the projects (not shown in the table), but a radical change for projects with a large number of classes. With this approach, the cost of maintaining the class hierarchy is assumable; in return, the retrieval of instances at whatever level is trivial.

The previous discussion is about uploading and downloading the entire ontology, a task that is performed during the development phase of a project. The user interaction with the system, adding and retrieving data, is no different from other systems. The user interaction involves only a small set of data, not the entire ontology.

OntoCRF demonstrates that an ontology-based approach is more flexible and efficient to deal with complexity and change than a traditional system, facilitating the engineering of clinical software systems. First of all, the application development phase is reduced to only analysis and design. The availability of prototypes from the very beginning, and the facility to apply changes, make OntoCRF an extremely useful tool to check the requirements and the solutions proposed. These facts imply a very important drop in costs and time with their consequent savings.

Secondly, differences between applications are reduced to their conceptual model. Therefore, the same infrastructure can be used for different projects, taking advantage of scale economy. All projects implemented until now share the same hard and soft infrastructure. The only difference between them is the content.

At the conceptual level, some elements or models can be reused in different projects, so homogeneous criteria and conceptual models could be established inside an organization. Concepts such as patient, clinical manifestations, and laboratory results are common in different projects, so these definitions can be easily shared and extended as needed.

The use of ontologies provides the ability to manage data structures declaratively, thus focusing the design on the conceptual aspects and not on the technical issues. Making an ontological analysis of an application allows for focus on a higher abstraction level and to concentrate on the domain aspects, thus helping researchers to clarify the implicit knowledge to manage. Moreover, the communication between designers and users is established at a conceptual level. Technical discussions that often contaminate the conceptual analysis in other approaches can be avoided. Moreover, ontologies assure that data and knowledge used in the project remain well documented.

Because the solution allows for modification of the underlying schema of the data, some measures are needed to guarantee the consistency of the instances. Problems could arise if trying to modify or delete classes or properties. The first security level is provided by Protégé, which does not allow performing some actions that could leave the ontology in an inconsistent state. This is the case when trying to delete a class that has instances. The rest of cases should be solved by the specification of editorial policies. When a project is running, deleting a class or property could be replaced by setting a deprecated flag on the resource. Nevertheless, in the database data are never physically deleted, only a delete flag is used to prevent the loss of data by mistake.

We consider the use of OWL and Protégé a good choice. The expressivity power of the language was adequate to cover the requirements of the projects in which OntoCRF was used. Moreover, it eases the interchange and reuse of models. The use of OWL allows adding reasoning capabilities in the future, a very promising line to explore.

From the usability study, it can be concluded that OntoCRF is well accepted by nearly 60% of its users, who considered the solution globally above average. But in a more detailed look at the data, high fragmentation is shown resulting in 4 groups with a very different perception of usability, from the best grade of “excellent” to the worst as “fail.” One explanation for such discrepancy could be a misunderstanding of the product under evaluation. OntoCRF has 2 components: a portal (developed using Liferay) customizable by the administrator of each community, and a database access for collecting the data. Moreover, OntoCRF is conceived as a full service in the cloud. Therefore, many different factors and user experiences can be interposed in the routine operation. The SUS score was developed in 1986, when many software solutions were developed for mainframe use or in a client-server environment. At present, widespread Internet usage interposes many more layers between the user interface and the physical data repositories. In this scenario, we need to better inform the users about what is being measured with the SUS score tool and perhaps develop new tools better suited for such new systems architecture. Nevertheless, further usability studies are required to improve OntoCRF, including specific questions with better information about the reasons of a low grading by some users.

Although the system is primarily used in health-related projects, the model is totally independent of the domain, so it would be suitable to gather data in any context. In fact, some projects implemented with OntoCRF are not about clinical information, but about management-related data. In general, if it is possible to model the data with OWL, it is possible to use OntoCRF.

### Limitations

We are aware of the system limitations. The metamodel of OntoCRF is not capable of process representation; hence, it is not able to manage explicit knowledge related to processes at the moment. The data extraction capacity is also limited. Currently, the final user cannot perform direct consultations over the server. Instead, data need to be previously extracted. This limitation is currently being addressed and some tools are being tested with the aim to be integrated with OntoCRF.

### Comparison With Prior Work

OntoCRF proposes the use of ontologies to ease and speed up the development of data repositories. The ontology-driven development of complex and intelligent systems has been largely applied in the past, especially when the ontologies or the methods are likely to be reused for new or derivative applications [16,39,40]. In general, the goal is to transform the system development cycle, so instead of programming each new application from scratch, we can select, modify, and assemble existing components [41]. Ontologies are used to build knowledge bases containing detailed descriptions of particular application areas. OntoCRF goes a step further because there is no need for programming, just the design of the application ontology. OntoCRF ontologies contain not only knowledge about the domain, but also the detailed description of the application.

The discussed approach is also related to the model-driven architecture (MDA) launched by the Object Management Group (OMG) [42]. According to their manifesto, MDA is a style of enterprise application development and integration based on using automated tools to build system-independent models and transform them into efficient implementations. As with ontology-oriented approaches, software evolution is handled simply by editing the underlying model. OMG is guided to object-oriented applications, particularly to distributed ones. It represents a more technical approach, centered on the platform independence, whereas OntoCRF pursues the conceptual independence. In our case, the database never changes and neither does the implementation of the application. Our work represents an advance because everything is defined explicitly, but the use is much more restricted.

In regard to research in data repositories, there exist multiple ontology-driven solutions for discovering and searching existing resources [43,44] or to consolidate clinical research data from disparate databases [4], but not much for automatically building new ones.

Compared with Protégé, WebProtégé [45] adds collaboration support and improves knowledge acquisition, but remains primarily an ontology editor. The work of Li et al [12] is close to our work in considering ontologies as the center of the architecture. The proposed system is focused on modeling a domain and supporting data and model changes, through versioning and dynamic composition, while using a simple interface with few options. On the other hand, Butt et al [46] propose the automatic generation of Web forms from ontologies with the objective of facilitating the creation of RDF data. Although the system produces easy-to-use forms, the capabilities of structuring the information are very limited.

As of this writing and to the best of our knowledge, there are no frameworks allowing the creation of data repositories, with the interface functionalities of traditional systems, in such a dynamic way such as OntoCRF, where even the user interface is built through the edition of ontologies.

There exist several works regarding how to store RDF graphs and ontologies, such as triple stores or relational databases that may be accessed as RDF graphs. However, none of these fulfill the needs of our system. In the case of RDF-based access to relational databases, such as the platform D2RQ [47], the system is read-only and just provides a RDF view of the content, but it does not provide any solution for storing the content, instead relying on an existing database created by the user. Also, the user has to generate the mappings between the platform and the database, specific for each use case. In the case of triple stores, they offer a way to store and retrieve triples, leaving the logic necessary for interpreting the triples and retrieving the right ones to an API or a query engine. This requires analyzing the whole set of triples of a specific graph, which has a high cost and is not scalable to big ontologies.

Our repository is not the only one with these characteristics, although it was at the time of our search for solutions. Systems such as OWLIM [48] and DLDB2 [49] combine DBMSs with additional capabilities for partial OWL reasoning. Furthermore, there are repositories with similar architectures to ours, such as Minerva [50] repository within the Integrated Ontology Development Toolkit by IBM. Because OntoDDB does not have Simple Protocol and RDF Query Language (SPARQL) capability yet, we could not perform any reliable comparison under equivalent conditions to these similar repositories.

### Future Work

We are considering different lines for future work. As previously mentioned, we are currently working on the integration of existing data query tools with OntoCRF to provide query functionalities to the final user. We plan to include SPARQL in the following months.

In a different line of work, we plan to use OntoCRF as the framework to build new electronic medical record (EMR) systems semantically interoperable. OWL representation provides an environment to integrate information models and terminology models used in the clinical context [35]. Currently, we have a prototype which implements the standard ISO 13606 in a native way, and there is ongoing work to conform to the standard EN 13940. These solutions were built using OntoCRF.

Finally, promising research work is being done to use existing ontologies and tools more intensively. Currently, ontologies are being used in OntoCRF as a data-modeling tool, so the use of already existing ontologies is a natural step. Moreover, applying automatic reasoning to data gathered in a project and integrated with external ontologies could provide interesting benefits.

### Conclusions

OntoCRF is a complete framework to build data repositories because it includes design of the system, storage, and GUI. The combination of ontologies and relational technology provides a system that is both flexible and solid. The ontology-based approach is more flexible and efficient to deal with complexity and change than traditional systems. On the other hand, storing the data in a relational database provides the known advantages of a solid relational model.

Although the GUI was not among our priorities, most participants of our usability study computed a global SUS score over 68, which is recognized as above average.

OntoCRF does not require very skilled technical people to make a new project, easing the engineering of clinical software systems. Moreover, the reduction of the development phase implies an important drop in costs and time. Furthermore, because the same infrastructure can be used for different projects, there is no need to dedicate specific equipment for each new project.

At the conceptual level, the ontological analysis of applications allows for concentration on the domain aspects, helping researchers to clarify the implicit knowledge to manage and to facilitate the communication between designers and users. Because some concepts are common in different projects, the models can be reused. On the other hand, ontologies assure that data and knowledge used in the project remain well documented. In addition, OWL and Protégé have proven enough expressivity to cover the requirements of the projects in which OntoCRF was used.

Finally, although currently the system is primarily used in health-related projects, the model is independent of the domain and can be useful in any project in which a distributed collection of data is needed.

## Abbreviations

API: application programming interface
CAPS: catastrophic antiphospholipid syndrome
DBMS: database management system
EAV: Entity-Attribute-Value
EMR: electronic medical record
EPR: electronic patient record
GUI: graphical user interface
ICT: information and communication technologies
IP: Internet Protocol
IRI: internationalized resource identifiers
JSTL: JSP Standard Tag Library
MDA: model-driven architecture
MVC: model-view-controller
OMG: Object Management Group
OntoCRF: Onto Clinical Research Forms
OntoDDB-MM: ontology-driven database metamodel
OWL-DB: OWL database
OWL: Ontology Web Language
RDF: Resource Description Framework
SPARQL: Simple Protocol and RDF Query Language
SUS: System Usability Scale
